# Complete genome sequence of *Serratia nematodiphila* G5.M166, an antifungal soil isolate from Hong Kong

**DOI:** 10.1128/mra.00912-25

**Published:** 2025-11-06

**Authors:** C. H. N. Ng, K. M. Leung, G. K. K. Lai, S. D. J. Griffin

**Affiliations:** 1 Shuyuan Molecular Biology Laboratory, The Independent Schools Foundation Academy 257202 , Hong Kong, China; California State University San Marcos, San Marcos, California, USA

**Keywords:** *Serratia nematodiphila*, *Aspergillus oryzae*, antifungal activity, soil

## Abstract

The complete genome of *Serratia nematodiphila* G5.M166, a soil isolate from Hong Kong with antifungal properties, was established through hybrid assembly. It has a single circular chromosome with a genome size of 5,325,120 bp (59.49% G+C).

## ANNOUNCEMENT

Characteristic of the *Serratia marcescens* complex, *Serratia nematodiphila* has been identified with infection (1, 2) but is nevertheless not among hospital-adapted lineages (3). More often, the species has been isolated from the intestines of invertebrates (4–7), from plants (8), and from soil (9). Strains of *S. nematodiphila* promote plant growth via phosphate solubilization, plant hormone production, drought, and heavy metal tolerance (8–11), as well as through their insecticidal and antifungal activity (12, 13). Its environmental resilience, particularly in the presence of heavy metals, has even been exploited in the synthesis of nanoparticles (14). Here, the soil isolate *S. nematodiphila* G5.M166 was found antagonistic toward *Aspergillus oryzae*.

A 1 g sample of soil collected from Pak Lap, Hong Kong (22.351833N, 114.356167E; 2022-11-04) was vortexed in 19 mL saline (0.9% [wt/vol]), and the mixture was allowed to settle for 5 min. After a further 1,000-fold dilution, a 100 µL aliquot was spread onto Luria agar (15) and incubated for 48 h. While this medium favors bacterial growth, filamentous fungi were numerous enough to observe the inhibition of hyphal growth around a single red colony of G5.M166. This colony was passaged to purity on Luria agar, and its antifungal activity against *Aspergillus oryzae* was confirmed by dual culture on rice (16) and on SDA agar (17), incubated for 72 h. A single colony of G5.M166 was subsequently streaked onto Luria agar to obtain overnight growth for DNA extraction using the DNeasy PowerSoil Pro Kit (QIAGEN GmbH). All agar incubations were at 27°C.

Paired-end short-read libraries (NEBNext Ultra DNA Library Prep Kit, Illumina, Inc.) were sequenced via the NovaSeq 6000 platform using an SP PE250 flowcell and v1.5 Reagent Kit. A total of 3,739,950 raw reads (mean length 250 bp) (SRX30105555) were quality-filtered and trimmed using TrimGalore! v0.6.7 (https://github.com/FelixKrueger/TrimGalore) (stringency:3; -e:0.2), producing 1,866,637 read pairs (mean length 248 bp, total 463 Mbp). Long-read libraries, prepared from the same extracted DNA using the Rapid Barcoding Kit SQK-RBK004 without size selection, were sequenced on Oxford Nanopore’s Spot-ON Flow Cell (vR9.4.1), MinION sequencer using MinKNOW v20.06.2 software, with basecalling by Guppy v6.1.55 (high-accuracy mode). The final long-read data set of 324,192 raw reads (mean length 3,838 bp, N50 6,368) (SRX30105556) was trimmed by Filtlong v0.2.1 (https://github.com/rrwick/Filtlong) (min_length:2000; target_bases:500Mbp; length_weight:10), selecting 88,309 reads (mean length 8,744 bp, N50 9,035; total 772 Mbp) to scaffold the assembly. Default parameters were used for all software unless otherwise specified.

Hybrid assembly of NovaSeq and MinION filtered data sets, overlap removal, and sequence rotation were performed using Unicycler v0.5.0 (18), yielding a single circular chromosome (5,325,120 bp, 59.49% G + C, mean coverage 146×; rotated to begin *dnaA*), which was submitted to NCBI PGAP v6.10 (19) for annotation. JSpecies v5.0.2 (20) found G5.M166 to share an average nucleotide identity (ANIb by BLAST+ v2.11.0 [21]) of 98.00% with type strain *Serratia nematodiphila* DSM 21420 (GCF_000738675.1).

Chromosomal genes linked to antifungal activity, as predicted by NCBI PGAP v6.10 or identified by BLAST in Uniprot v2025_03 (22) and by SecReT6 v3 (23), are shown in Table 1.

**TABLE 1:** Genes in G5.M166 potentially linked to antifungal activity, as predicted by NCBI PGAP v6.5 (19), with additional elucidation by BLAST in Uniprot v2025_03 (22) (*) and by SecReT6 v3 (23) (**)^*a*^

Gene(s)	NCBI locus	Activity	Reference
*chiA *chiB *chiC chbG	ACRA7N_00425 ACRA7N_18680 ACRA7N_06155 ACRA7N_03865	Chitinases A, B, and C, EC 3.2.1.14; chitin disaccharide deacetylase, EC 3.5.1.105	(16, 23, 24)
cueR-*pigABCDEF- *pigGHIJKLMN-copA	ACRA7N_05390 to ACRA7N_05315	Prodigiosin synthesis	(25–27)
**T6SS region 1	ACRA7N_15615 to ACRA7N_15845	Type VI secretion system: delivers toxic effector proteins into targeted fungal cells	(28, 29)
**T6SS region 2	ACRA7N_16380 to ACRA7N_16505		
*swrW	ACRA7N_23195	Putative serrawettin W1 synthetase	(27)
*entB	ACRA7N_01375	Enterobactin biosynthesis: isochorismatase, EC 3.3.2.1	(30)
*hcnC	ACRA7N_09215	Hydrogen cyanide synthase subunit HcnC	(30)
budA	ACRA7N_18460	Acetoin biosynthesis: alpha-acetolactate decarboxylase, EC 4.1.1.5	(30)

^*a*^ Uniprot BLAST data is available at https://doi.org/10.6084/m9.figshare.30261607; T6SS predictions are at https://doi.org/10.6084/m9.figshare.30273802.

## Data Availability

Sequencing data for *Serratia nematodiphila* G5.M166 is available through NCBI under GenBank assembly GCA_050845915.1 and GenBank accession CP193483. Raw reads may be found under SRX30105555 (NovaSeq) and SRX30105556 (MinION). BLAST data via Uniprot and SecReT6 available at https://doi.org/10.6084/m9.figshare.30261607 and https://doi.org/10.6084/m9.figshare.30273802 respectively.
